# Association of metabolic dysfunction-associated fatty liver disease with chronic kidney disease: a Chinese population-based study

**DOI:** 10.1080/0886022X.2022.2144373

**Published:** 2022-11-14

**Authors:** Qian Hu, Yao Chen, Ting Bao, Yan Huang

**Affiliations:** a Health Management Center, West China Hospital of Sichuan University, Chengdu, China; b Department of Breast Surgery, West China Hospital, Sichuan University, Chengdu, China

**Keywords:** Metabolic dysfunction-associated fatty liver disease, chronic kidney disease, diabetes, hyperuricemia, remnant cholesterol

## Abstract

**Background:**

Metabolic dysfunction-associated fatty liver disease (MAFLD) is a multisystem disorder, but its relationship with kidney injury remains controversial. This study aimed to evaluate MAFLD effects on the chronic kidney disease (CKD) prevalence in a general population in China.

**Methods:**

In total, 15,010 individuals from the Health Management Center of West China Hospital from July 2020 to June 2021 were screened. Hepatic steatosis was defined as a median FibroScan controlled attenuation parameter (CAP)≥240 dB/m using liver ultrasound transient elastography. CKD was defined as an estimated glomerular filtration rate (eGFR) <60 mL/min/1.73 m^2^ and/or the presence of albuminuria. The association of MAFLD with CKD was examined using logistic regression. Risk factors for CKD in different MAFLD subgroups were also investigated.

**Results:**

A total of 8226 individuals were finally included. Of them, 4406 (53.6%) had MAFLD, and 592 (7.2%) had CKD. After propensity score matching (PSM), 5530 eligible subjects were selected (*n* = 2765 in each group). There was a higher CKD prevalence in subjects with MAFLD than in those without MAFLD (8.9% vs. 5.4%, *p* < 0.001). MAFLD was significantly associated with a higher CKD prevalence (OR 1.715, 95% CI 1.389–2.117, *p* < 0.001), although it was not an independent risk factor. The results indicated that age, diabetes mellitus (DM), overweight/obesity, hypertension, hyperuricemia, hypertriglyceridemia, remnant cholesterol (RC), and C-reactive protein (CRP) were independently associated with a higher CKD prevalence. In the subgroup analysis, hypertension, hyperuricemia, RC, and the nonalcoholic fatty liver disease fibrosis score (NFS) were independent risk factors for the prevalence of CKD in individuals with DM or prediabetes and MAFLD. Furthermore, hypertension, hyperuricemia, and body fat percentage (BFP) were independently associated with CKD in subjects with MAFLD without DM.

**Conclusion:**

Individuals with MAFLD had a higher prevalence of CKD, whereas it was not an independent risk factor for CKD.

## Introduction

Nonalcoholic fatty liver disease (NAFLD) has become the most common cause of chronic liver disease and is characterized by accumulated fat in liver cells [1]. The prevalence of NAFLD has reached an alarming proportion, affecting nearly 25% of adults worldwide and 30% of adults in China [2]. Emerging evidence has established relationships between NAFLD and chronic kidney disease (CKD), cardiovascular disease (CVD), and diabetes [3,4].

Metabolic dysfunction-associated fatty liver disease (MAFLD), a new condition proposed by an international expert consensus in 2020 [5], better characterizes the role of metabolic disorders in fatty liver disease than NAFLD. Its diagnosis is based on three manifestations, including overweight/obesity, type 2 diabetes mellitus (T2DM) or metabolic dysfunction. Hence, the major benefit of the MAFLD classification is that it reflects the notion that multiple metabolic factors promote hepatic steatosis and do not simply just coexist [6]. Therefore, early identification of those at high risk of metabolic disorders may contribute to the further prevention of MAFLD.

CKD has become a major health-threatening disease in the twenty-first century, affecting 15% of individuals worldwide; moreover, CKD gradually progresses to end-stage renal disease (ESRD), which imposes a substantial burden on human health and the social economy [7]. Accordingly, there is an urgent need to identify more specific biomarkers and search for new CKD interference targets. Previous studies have demonstrated that hypertension, DM, hyperuricemia, and obesity are associated with an increased risk of CKD [8]; however, whether MAFLD can differentiate individuals at high risk of CKD is still uncertain.

In this study, we aimed to evaluate the association between MAFLD and CKD in a Chinese population and investigate the risk factors for CKD in individuals with MAFLD.

## Methods

### Study population

**Figure 1.**
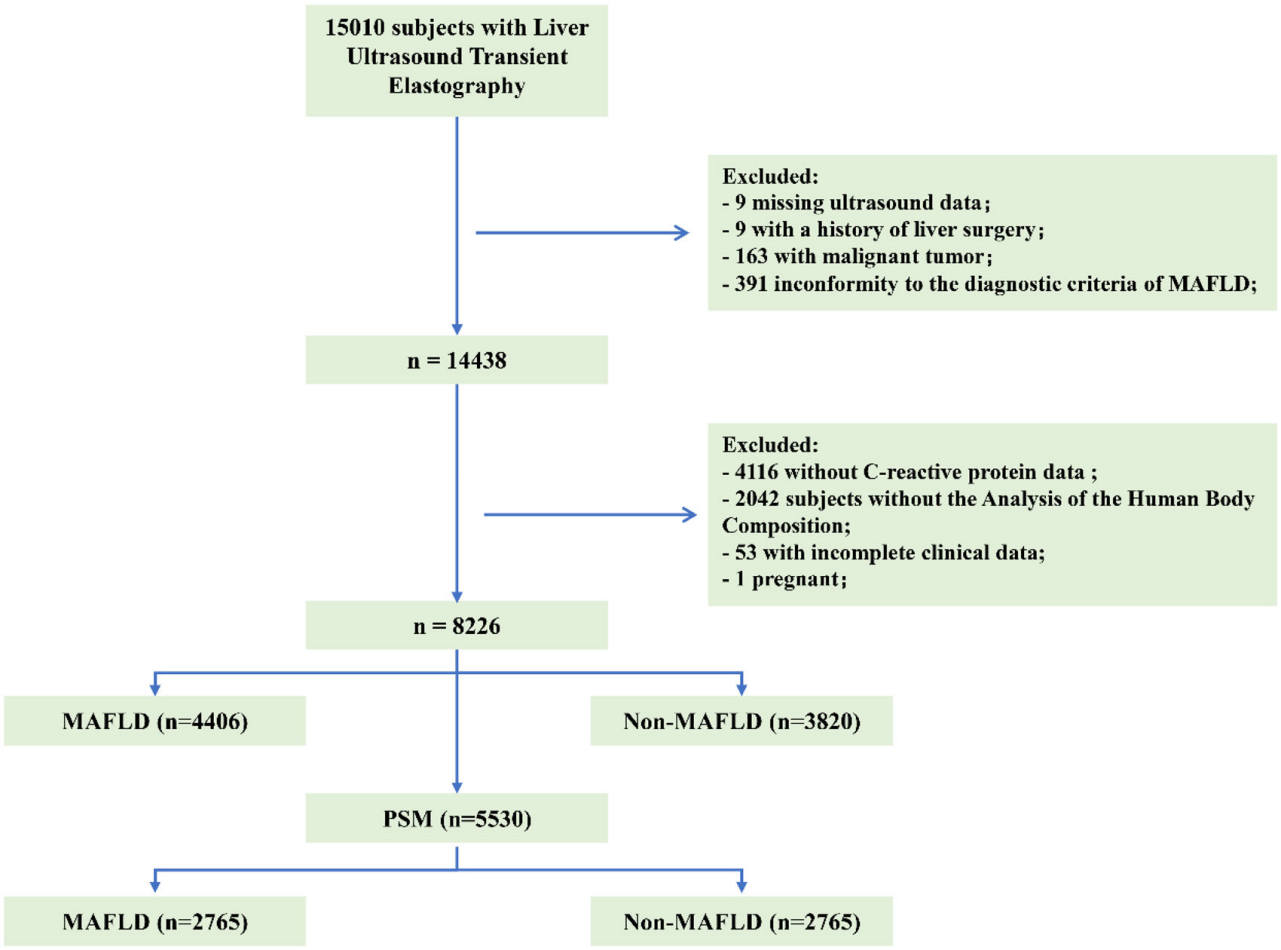
The flowchart of the subjects included in this study. PSM: Propensity Score Matching.

This cross-sectional study analyzed the data of 15,010 Chinese individuals who underwent liver ultrasound transient elastography examinations at the Health Management Center of West China Hospital from July 2020 to June 2021. Of them, 6784 were excluded for the following reasons: (1) missing ultrasound data, (2) a history of liver surgery, (3) presence of a malignant tumor, (4) nonconformity to the diagnostic criteria of MAFLD [5], (5) missing C-reactive protein (CRP) data, (6) missing body composition analysis data, (7) incomplete clinical data, or (8) pregnancy (Figure 1). The inclusion criteria were as follows: (1) age ≥18 years old, (2) a diagnosis of MAFLD, and (3) comprehensive health checkup data. The related general information and clinical data of all eligible individuals were collected. Hepatic steatosis was defined as a median FibroScan controlled attenuation parameter (CAP) ≥240 dB/m using liver ultrasound transient elastography. Body composition analysis was performed with an InBody analyzer (InBody570) based on bioelectrical impedance technology. This study was approved by the Ethics Committee of West China Hospital of Sichuan University, and informed consent was obtained from all subjects.

### Diagnostic criteria for MAFLD

The established 2020 criteria for MAFLD include evidence of hepatic steatosis plus one of the following: overweight/obesity, type 2 DM, or the presence of metabolic disorder. Metabolic disorder was defined as evidence of no less than two of the following metabolic risk factors: (1) waist circumference ≥102/88 cm in Caucasian men and women (or ≥90/80 cm in Asian men and women); (2) blood pressure ≥130/85 mmHg or related drug treatment; (3) plasma triglycerides ≥150 mg/dl or related drug treatment; (4) plasma high-density lipoprotein cholesterol (HDL-C) <40 mg/dl in men and <50 mg/dl in women or related drug treatment; (5) prediabetes (i.e. fasting glucose level of 100–125 mg/dl, or 2-h post-load glucose level of 140–199 mg/dl or HbA1c 5.7–6.4%); (6) homeostasis model assessment of insulin resistance (HOMA-IR) score ≥2.5; and (7) plasma high-sensitivity CRP level >2 mg/L [5]. Overweight or obesity was defined as body mass index (BMI) ≥25 kg/m^2^ in Caucasians or BMI ≥23 kg/m^2^ in Asians. Diabetes mellitus (DM) in this study was defined as a history of diabetes or HbA1c ≥6.5% [9], and prediabetes was defined as a level of HbA1c of 5.7–6.4%.

### Other diagnostic criteria

CKD was defined as an estimated glomerular filtration rate (eGFR) <60 mL/min/1.73 m^2^ (CKD-EPI [10]) and/or the presence of albuminuria, defined as a urinary albumin-to-creatinine ratio (UACR) ≥30 mg/g. Hypertension was defined as a history of hypertension or blood pressure ≥140/90 mmHg. Hyperuricemia was defined as a blood uric acid level ≥420/360 μmol/L in men and women. Remnant cholesterol was defined as total cholesterol (mmol/L) – low-density lipoprotein cholesterol (LDL-C) (mmol/L) – HDL-C (mmol/L) [11]. The fibrosis 4 (FIB-4) score and NAFLD fibrosis score (NFS) were used to assess the degree of liver fibrosis. Their formulas are as follows: FIB-4 = age (years)×AST(IU/L)/(PLT(10^9^/L)×ALT(IU/L)^1/2^), NFS= −1.675 + 0.037 × age(years)+0.094 × BMI(kg/m^2^)+1.13 × impaired fasting glucose/diabetes (yes = 1, no = 0) +0.99 × AST/ALT − 0.013× platelet (×10^9^/l) − 0.66 × albumin (g/dl) [12].

### Data collection

The demographic and clinical information of subjects was collected at the time of physical checkup and included the following: (1) age, sex, ethnicity, height, weight, systolic/diastolic blood pressure, medical history, waist circumference, etc.; (2) HbA1c, blood uric acid, serum creatinine, eGFR, UACR, etc.; (3) LDL-C, HDL-C, alanine aminotransferase (ALT), aspartate aminotransferase (AST), alkaline phosphatase (ALP), gamma-glutamyl transpeptidase (GGT), etc.; and (4) CRP, body fat percentage (BFP) (overall body fat/body weight), basal metabolic rate, etc.

### Statistical analysis

All calculations were performed using SPSS software (version 26.0, IBM Corp., Armonk, NY, USA). The included individuals were divided into two groups, namely, the MAFLD and non-MAFLD groups. Propensity score matching (PSM) with a caliper of 0.02 was used to match the following three variables in a 1:1 ratio between two groups: age, sex, and ethnicity. Continuous variables are expressed as means ± standard deviations (SDs) or medians with interquartile ranges (IQRs), and categorical data are presented as frequencies and percentages. The normality of the data was assessed using the Kolmogorov–Smirnov test. Differences between the two groups were calculated by Student’s *t* test, the Mann–Whitney *U* test, or the chi-square test, as appropriate. Logistic regression analysis was performed to identify risk factors for CKD. A two-sided *p* < 0.05 was considered statistically significant.

## Results

### Characteristics of the participants

A total of 8226 individuals were included in this study (Figure 1), with 4406 in the MAFLD group and 3820 in the non-MAFLD group. Of them, 592 (7.2%) had CKD, 704 (8.5%) had DM, 2770 (33.7%) had prediabetes and 5406 (65.7%) were overweight or obese. As shown in Table 1, before PSM, CKD was more prevalent in the MAFLD group than in the non-MAFLD group (9.3 vs. 4.8%, *p* < 0.001). This difference was maintained after PSM (8.9% in the MAFLD group vs. 5.4% in the non-MAFLD group, *p* < 0.001). Age and the percentage of males were significantly different between the two groups but were balanced after PSM.

**Table 1.** Baseline characteristics before and after propensity score matching (PSM).

Variates	Before PSM			After PSM		
	Non-MAFLD (*n* = 3820)	MAFLD (*n* = 4406)	*p* value	Non-MAFLD (*n* = 2765)	MAFLD (*n* = 2765)	p* value
Age (years)	47 (38-54)	42 (49-55)	<0.001	48.90 ± 10.32	49.04 ± 10.43	0.61
Male (*n*, %)	1671 (43.7)	3266 (74.1)	<0.001	1670 (60.4)	1667 (60.3)	0.956
Chinese Han (*n*, %)	3678 (96.3)	4214 (95.6)	0.142	2653 (95.9)	2639 (95.4)	0.389
DM (*n*, %)	148 (3.9)	556 (12.6)	<0.001	143 (5.2)	337 (12.2)	<0.001
Prediabetes (*n*, %)	1019 (26.7)	1751 (39.7)	<0.001	866 (31.3)	1137 (41.4)	<0.001
HbA1c (%)	5.5 (5.3-5.7)	5.7 (5.4-6)	<0.001	5.5 (5.3-5.8)	5.7 (5.4-6)	<0.001
Hypertension (*n*, %)	647 (16.9)	1189 (27)	<0.001	534 (19)	763 (28)	<0.001
Smoking (*n*, %)	1002 (26.2)	1756 (39.9)	<0.001	941 (34)	872 (32)	0.051
Overweight/obesity (*n*, %)	1375 (36)	4031 (91.5)	<0.001	1173 (42.4)	2479 (89.7)	<0.001
BMI (kg/m^2^)	22.06 (20.43-23.78)	25.89 (24.34-27.7)	<0.001	22.52 (20.83-24.17)	25.69 (24.13-27.55)	<0.001
Waist circumference (cm)	76 (70-82)	89 (83-94)	<0.001	78.41 ± 8.471	87.54 ± 8.59	<0.001
Body fat percentage (%)	0.269 (0.228-0.314)	0.303 (0.266-0.345)	<0.001	0.26 (0.22-0.31)	0.32 (0.27-0.36)	<0.001
Basal metabolic rate (kcal)	1303.22 ± 172.78	1455.19 ± 184.52	<0.001	1351 (1191-1484)	1421 (1243-1553)	<0.001
CKD (*n*, %)	182 (4.8)	410 (9.3)	<0.001	149 (5.4)	246 (8.9)	<0.001
Albuminuria (*n*, %)	151 (4.0)	386 (8.8)	<0.001	118 (4.3)	233 (8.4)	<0.001
eGFR (ml/min/1.73 m^2^)	97.03 (86.65-105.86)	94.29 (84.01-102.77)	<0.001	92.80 ± 13.96	93.69 ± 13.85	0.016
eGFR categories			<0.01			0.093
≥90 (*n*, %)	2571 (67.3)	2698 (61.2)		1677 (60.7)	1753 (63.4)	
60-90 (*n*, %)	1204 (31.5)	1660 (37.7)		1044 (37.8)	983 (35.6)	
30-60 (*n*, %)	41 (1.1)	46 (1.0)		40 (1.4)	28 (1.0%)	
15-30 (*n*, %)	2	1		2	0	
<15 (*n*, %)	2	1		2	1	
Serum creatinine (μmol/L)	72 (61-84)	81 (70-90)	<0.001	78 (65-88)	77 (64-88)	0.025
Hyperuricemia (*n*, %)	468 (12.3)	1465 (33.3)	<0.001	419 (15.2)	832 (30.1)	<0.001
Hypertriglyceridemia (*n*, %)	675 (17.7)	2357 (53.5)	<0.001	606 (21.9)	1425 (51.5)	<0.001
Total cholesterol (mmol/L)	4.85 ± 0.91	5.05 ± 0.96	<0.001	4.84 (4.27-5.47)	5.01 (4.41-5.61)	<0.001
HDL-C (mmol/L)	1.41 (1.19-1.68)	1.12 (0.96-1.33)	<0.001	1.35 (1.14-1.61)	1.15 (0.98-1.37)	<0.001
LDL-C (mmol/L)	2.86 (2.37-3.42)	3.09 (2.58-3.61)	<0.001	2.94 (2.44-3.49)	3.09 (2.57-3.61)	<0.001
Remnant cholesterol (mmol/L)	0.41 (0.29-0.57)	0.63 (0.46-0.88)	<0.001	0.45 (0.32-0.62)	0.62 (0.45-0.86)	<0.001
CRP (mg/L)	1.75 (1.36-2.52)	2.37 (1.73-3.54)	<0.001	1.81 (1.41-2.59)	2.36 (1.73-3.55)	<0.001
ALT (IU/L)	17 (12-24)	26 (18-38)	<0.001	18 (13-26)	24 (17-36)	<0.001
AST (IU/L)	20 (17-24)	23 (19-28)	<0.001	21 (18-25)	22 (19-28)	<0.001
ALP (IU/L)	70.96 ± 21.50	77.27 ± 20.86	<0.001	74.21 ± 21.29	78.11 ± 21.60	<0.001
GGT (IU/L)	16 (12-27)	32 (20-55)	<0.001	19 (13-31)	29 (18-51)	<0.001
FIB-4 score	0.88 (0.58-1.32)	1.31 (0.91-1.89)	<0.001	1.00 (0.70-1.47)	1.25 (0.86-1.25)	<0.001
FIB-4 category			<0.001			<0.001
FIB-4 < 1.3 (Grade 1)	2838 (74.3)	2180 (49.5)		1885 (68.2)	1445 (52.3)	
1.3 ≤ FIB-4 < 2.67 (Grade 2)	832 (21.8)	1790 (40.6)		741 (26.8)	1070 (38.7)	
FIB-4 ≥ 2.67 (Grade 3)	150 (3.9)	436 (9.9)		139 (5.0)	250 (9.0)	
NFS score	−2.32 (-3.06 to 1.58)	−2.11 (-2.94 to 1.31)	<0.001	−2.17 (2.93 to 1.41)	−2.10 (2.97 to 1.24)	0.014
NFS category			<0.001			0.001
NFS<-1.455 (Grade 1)	2972 (77.8)	3135 (71.2)		2049 (74.1)	1933 (69.9)	
−1.455 ≤ NFS < 0.676 (Grade 2)	821 (21.5)	1220 (27.7)		693 (25.1)	794 (28.7)	
NFS ≥ 0.676 (Grade 3)	27 (0.7)	51 (1.2)		23 (0.8)	38 (1.4)	

Data are presented as the mean ± SD, the median with interquartile range, or counts and percentages.

DM: diabetes mellitus; HbA1c, glycosylated hemoglobin; BMI: body mass index; eGFR: estimated glomerular filtration rate; CKD: chronic kidney disease; ALP: alkaline phosphatase; ALT: alanine aminotransferase; AST: aspartate aminotransferase; GGT: γ-glutamyl transpeptidase; FIB-4: fibrosis-4; HDL-C: high-density lipoprotein cholesterol; LDL-C: low-density lipoprotein cholesterol; CRP: C-reactive protein; NFS: NAFLD fibrosis score.

*A two-tailed *p* < 0.05 was considered statistically significant.

Before PSM, the MAFLD group had a higher HbA1c level; higher BMI; larger waist circumference; higher BFP; higher basal metabolic rate; higher serum creatinine, total cholesterol, LDL-C, remnant cholesterol, CRP, ALT, AST, ALP, GGT levels; higher FIB-4 scores and NFS; lower eGFR; lower HDL-C level; and higher prevalence rates of CKD, DM, prediabetes, smoking, overweight/obesity, albuminuria, hyperuricemia, and hypertriglyceridemia than the non-MAFLD group.

After PSM, the MAFLD group still had a higher HbA1c level; higher BMI; larger waist circumference; higher BFP; higher basal metabolic rate; higher total cholesterol, LDL-C, remnant cholesterol, CRP, ALT, AST, ALP, and GGT levels; higher FIB-4 score and NFS; lower HDL-C level; and higher prevalence rates of CKD, DM, prediabetes, overweight/obesity, albuminuria, hyperuricemia, and hypertriglyceridemia than the non-MAFLD group. In contrast, after PSM, the MAFLD group had a higher eGFR and lower serum creatinine level than the non-MAFLD group. There were no differences observed in age, sex, ethnicity or smoking status between MAFLD and non-MAFLD groups.

### Association between MAFLD and CKD after PSM

**Figure 2.**
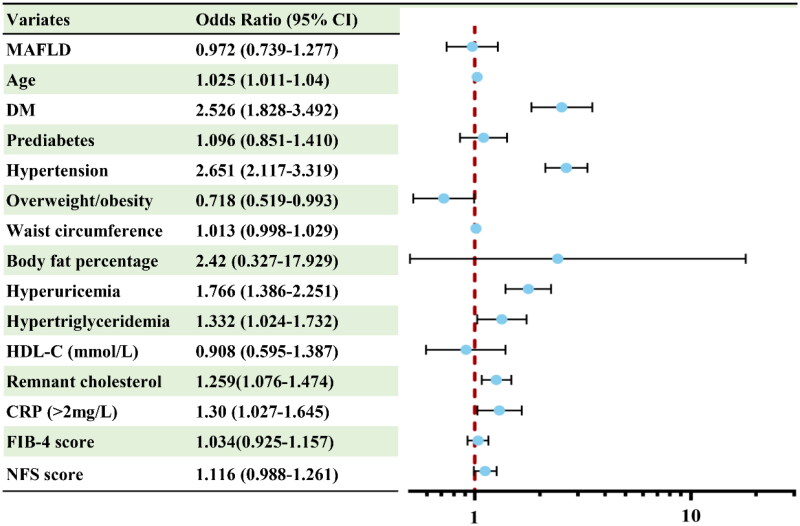
Risk factors for CKD based on the PSM using Logistic Regression analysis. Abbreviations: DM, diabetes mellitus; FIB-4, fibrosis-4; HDL-C, high-density lipoprotein cholesterol; CRP, C-reactive protein; NFS, NAFLD fibrosis score.

Logistic regression analysis was performed to reveal the relationship between MAFLD and CKD in subjects, as shown in Table 2. The univariate analyses showed that MAFLD, age, DM, prediabetes, hypertension, overweight/obesity, waist circumference, BFP, hyperuricemia, hypertriglyceridemia, HDL-C, remnant cholesterol, CRP, FIB-4 score, and NFS were significantly associated with an increased risk of CKD (*p* < 0.05). The covariates with a *p* value <0.05 in the univariate analyses were entered into the multivariate logistic regression model. After adjustment for the above covariates, age (OR 1.025, 95% CI 1.011–1.04, *p* = 0.001), DM (OR 2.526, 95% CI 1.828–3.492, *p* < 0.001), hypertension (OR 2.651, 95% CI 2.117–3.319, *p* < 0.001), overweight/obesity (OR 0.718, 95% CI 0.519–0.993, *p* = 0.045), hyperuricemia (OR 1.766, 95% CI 1.386–2.251, *p* < 0.001), hypertriglyceridemia (OR 1.332, 95% CI 1.024–1.732, *p* = 0.033), remnant cholesterol (OR 1.259, 95% CI 1.076–1.474, *p* = 0.004), and CRP (OR 1.30, 95% CI 1.027–1.645, *p* = 0.029) remained significantly associated with CKD (Figure 2). However, MAFLD was not significantly associated with CKD in the multivariate-adjusted model, suggesting that it was not an independent risk factor for prevalent CKD.

**Table 2.** Risk factors for CKD based on the PSM using Logistic Regression analysis.

Variates	Univariate				Multivariate	
	Non-CKD (*n* = 5135)	CKD (*n* = 395)	Odds ratio (95% CI)	*p* value	Odds ratio (95% CI)	p*value
MAFLD (*n*, %)	2519 (49.1)	246 (62.3)	1.715 (1.389-2.117)	<0.001	0.972 (0.739-1.277)	0.837
Age (years)	48 (42-56)	54 (47-60)	1.047 (1.037-1.058)	<0.001	1.025 (1.011-1.04)	0.001
Male (*n*, %)	3092 (60.2)	245 (62)	1.078 (0.874-1.332)	0.478		
DM (*n*, %)	378 (7.4)	102 (25.8)	5.323 (4.046-7.004)	<0.001	2.526 (1.828-3.492)	<0.001
Prediabetes (*n*, %)	1857 (36.2)	146 (37)	1.551 (1.225-1.964)	<0.001	1.096 (0.851-1.410)	0.478
Hypertension (*n*, %)	1098 (21.4)	199 (50.4)	3.733 (3.031-4.597)	<0.001	2.651 (2.117-3.319)	<0.001
Smoking (*n*, %)	1687 (32.9)	126 (31.9)	0.957 (0.769-1.192)	0.697		
Overweight/obesity (*n*, %)	3362 (65.6)	290 (73.4)	1.457 (1.157-1.834)	0.001	0.718 (0.519-0.993)	0.045
Waist circumference (cm)	83 (76-89)	86 (79-94)	1.042 (1.031-1.053)	<0.001	1.013 (0.998-1.029)	0.098
Body fat percentage (%)	0.287 (0.243-0.334)	0.309 (0.261-0.353)	73.225 (15.12-354.627)	<0.001	2.42 (0.327-17.929)	0.387
Hyperuricemia (n, %)	1110 (21.6)	141 (35.7)	2.013 (1.621-2.499)	<0.001	1.766 (1.386-2.251)	<0.001
Hypertriglyceridemia (n, %)	1823 (35.5)	208 (52.7)	2.021 (1.645-2.482)	<0.001	1.332 (1.024-1.732)	0.033
HDL-C (mmol/L)	1.25 (1.05-1.5)	1.15 (0.95-1.42)	0.461 (0.332-0.640)	<0.001	0.908 (0.595-1.387)	0.656
LDL-C (mmol/L)	3.03 (2.5-3.55)	3.01 (2.45-3.67)	0.978 (0.861-1.110)	0.726		
Remnant cholesterol (mmol/L)	0.52 (0.37-0.73)	0.65 (0.45-0.95)	1.618 (1.415-1.850)	<0.001	1.259 (1.076-1.474)	0.004
CRP (>2mg/L)	2621 (51)	257 (65.1)	1.786 (1.442-2.212)	<0.001	1.30 (1.027-1.645)	0.029
FIB-4 score	1.11 (0.76-1.63)	1.38 (0.96-2.03)	1.28 (1.176-1.394)	<0.001	1.034 (0.925-1.157)	0.554
NFS score	−2.17 (-2.99—1.38)	−1.58 (-2.47—0.70)	1.510 (1.385-1.647)	<0.001	1.116 (0.988-1.261)	0.076

Data are presented as the mean ± SD, the median with interquartile range, or counts and percentages.

DM: diabetes mellitus; FIB-4: fibrosis-4; HDL-C: high-density lipoprotein cholesterol; LDL-C: low-density lipoprotein cholesterol; CRP: C-reactive protein; NFS: NAFLD fibrosis score.

*A two-tailed *p* < 0.05 was considered statistically significant.

**Figure 3.**
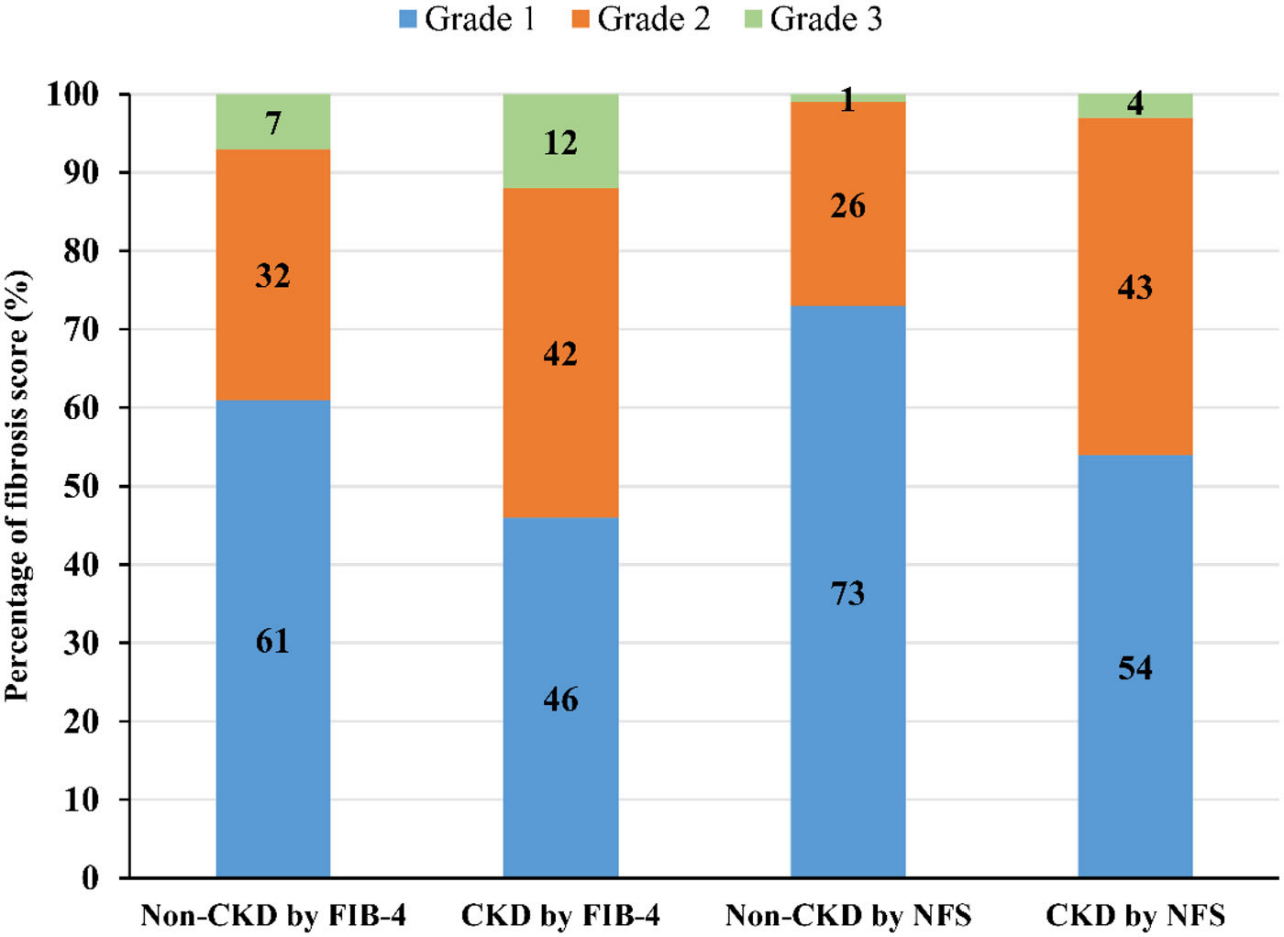
Fibrosis scores based on FIB-4 and NFS grades in CKD. CKD, chronic kidney disease; FIB-4, fibrosis-4; NFS, NAFLD fibrosis score.

Additionally, we explored the prevalence of different FIB-4 scores and NFS in subjects with and without CKD. As shown in Figure 3, FIB-4 scores of 1, 2, and 3 accounted for 46, 42, and 12%, respectively, in the CKD group and 61, 32, and 7%, respectively, in the non-CKD group. Moreover, NFS of 1, 2, and 3 accounted for 54, 43, and 4%, respectively, in the CKD group and 73, 26, and 1%, respectively, in the non-CKD group. Accordingly, the individuals with CKD had higher fibrosis scores than those without CKD (*p* < 0.001).

### Risk factors for CKD in subjects with DM or prediabetes and MAFLD

Among 1474 individuals with DM or prediabetes and MAFLD, 169 (11.47%) had CKD. The results of the multivariate logistic regression analysis indicated that hypertension (OR 3.429, 95% CI 2.401–4.896, *p* < 0.001), hyperuricemia (OR 1.495, 95% CI 1.035–2.158, *p* = 0.032), remnant cholesterol (OR 1.305, 95% CI 1.068–1.595, *p* = 0.009), and NFS (OR 1.359, 95% CI 1.141–1.617, *p* = 0.001) were independent risk factors for CKD after adjustment for age, overweight/obesity, waist circumference, BFP, hypertriglyceridemia, HDL-C, CRP, and the FIB-4 score (Table 3).

**Table 3.** Risk factors for CKD in DM or prediabetes subjects with MAFLD (169/1474).

Variates	Odds ratio (95% CI)	p* value
Age (years)	1.006 (0.983-1.029)	0.61
Hypertension	3.429 (2.401-4.896)	<0.001
Overweight/obesity	0.557 (0.292-1.062)	0.076
Hyperuricemia	1.495 (1.035-2.158)	0.032
Hypertriglyceridemia	1.189 (0.786-1.799)	0.413
Remnant cholesterol (mmol/L)	1.305 (1.068-1.595)	0.009
CRP (>2mg/L)	1.026 (0.691-1.524)	0.897
FIB-4 score	0.989 (0.835-1.171)	0.897
NFS score	1.359 (1.141-1.617)	0.001

CRP: C-reactive protein; FIB-4: fibrosis-4; NFS, NAFLD fibrosis score.

*A two-tailed *p* < 0.05 was considered statistically significant.

### Risk factors for CKD in subjects with DM or prediabetes without MAFLD

Among 1009 individuals with DM or prediabetes without MAFLD, 79 (7.83%) had CKD. The results of the multivariate logistic regression analysis suggested that age (OR 1.036, 95% CI 1.003–1.069, *p* = 0.034), hypertension (OR 2.393, 95% CI 1.447–3.959, *p* = 0.001), and hyperuricemia (OR 2.036, 95% CI 1.147–3.616, *p* = 0.015) were independently associated with CKD after adjustment for overweight/obesity, waist circumference, BFP, hypertriglyceridemia, HDL-C, CRP, the FIB-4 score, the NFS, and remnant cholesterol (Table 4).

**Table 4.** Risk factors for CKD in DM or prediabetes subjects without MAFLD (79/1009).

Variates	Odds ratio (95% CI)	p* value
Age (years)	1.036 (1.003-1.069)	0.034
Hypertension	2.393 (1.447-3.959)	0.001
Overweight/obesity	0.644 (0.34-1.223)	0.179
Hyperuricemia	2.036 (1.147-3.616)	0.015
Hypertriglyceridemia	1.804 (0.953-3.412)	0.07
Remnant cholesterol (mmol/L)	1.233 (0.629-2.42)	0.542
CRP (>2mg/L)	1.6 (0.974-2.626)	0.063
FIB-4 score	1.135 (0.907-1.421)	0.269
NFS score	1.139 (0.901-1.439)	0.276

CRP: C-reactive protein; FIB-4: fibrosis-4; NFS: NAFLD fibrosis score.

*A two-tailed *p* < 0.05 was considered statistically significant.

### Risk factors for CKD in subjects with MAFLD without DM

Among 2428 subjects with MAFLD without DM, 170 (7.0%) had CKD. The results of the multivariate logistic regression analysis showed that hypertension (OR 3.246, 95% CI 2.319–4.542, *p* < 0.001), hyperuricemia (OR 1.503, 95% CI 1.059–2.133, *p* = 0.023), and BFP (OR 34.849, 95% CI 1.549–784.001, *p* = 0.025) were independently associated with CKD after adjustment for age, overweight/obesity, waist circumference, hypertriglyceridemia, HDL-C, CRP, the FIB-4 score, the NFS, remnant cholesterol, and prediabetes (Table 5).

**Table 5.** Risk factors for CKD in MAFLD subjects without DM (170/2428).

Variates	Odds ratio (95% CI)	p* value
Age (y)	1.014 (0.993-1.036)	0.188
Hypertension	3.246 (2.319-4.542)	<0.001
Overweight/obesity	0.778 (0.431-1.403)	0.404
Hyperuricemia	1.503 (1.059-2.133)	0.023
Hypertriglyceridemia	1.225 (0.833-1.801)	0.303
Remnant cholesterol (mmol/L)	1.239 (0.952-1.612)	0.111
CRP (>2mg/L)	1.287 (0.89-1.862)	0.18
Prediabetes	1.073 (0.769-1.499)	0.677
Body fat percentage (%)	34.849 (1.549-784.001)	0.025
FIB-4 score	1.037 (0.876-1.228)	0.67
NFS score	1.09 (0.909-1.307)	0.353

CRP: C-reactive protein; FIB-4: fibrosis-4; NFS, NAFLD fibrosis score.

*A two-tailed *p* < 0.05 was considered statistically significant.

## Discussion

In this study, we retrospectively analyzed the relationship between MAFLD and CKD. A total of 8226 subjects met the inclusion criteria, and 592 (7.2%) subjects with CKD were included. Our results demonstrated that subjects with MAFLD had a higher prevalence of CKD than those without MAFLD before and after PSM. The logistic regression analysis indicated that MAFLD was significantly associated with CKD, although it was not an independent risk factor. The results also showed that age, diabetes, overweight/obesity, hypertension, hyperuricemia, hypertriglyceridemia, remnant cholesterol, and CRP were independent risk factors for CKD. We performed subgroup analyses and observed that hypertension, hyperuricemia, remnant cholesterol, and the NFS were independent risk factors for prevalence CKD in individuals with MAFLD and DM or prediabetes. Furthermore, hypertension, hyperuricemia, and BFP were independently associated with CKD in subjects with MAFLD without DM. In particular, hypertension and hyperuricemia were significantly associated with CKD in all subgroups.

The association of chronic renal injury with NAFLD has been extensively investigated over the years [13], but studies on the association of CKD with MAFLD are limited. The results of Deng et al. based on the dataset of the National Health and Nutrition Examination Survey (NHANES) 2017–2018 [12] in the United States, showed that MAFLD was not associated with CKD after adjustment for age, sex, and race, whereas the FIB-4 score was an independent risk factor for CKD. In addition, the study by Sun et al. based on the dataset of the NHANES 1988–1994, indicated that MAFLD was not independently associated with prevalent CKD (eGFR < 60 mL/min/1.73 m^2^, OR 1, 95% CI 0.89–1.13, *p* = 0.970) after adjustment for sex, age, ethnicity, alcohol intake, and preexisting diabetes [14].

In this study, we found that MAFLD was significantly related to the increased prevalence of CKD even after adjustment for age, sex and ethnicity, although it was not an independent risk factor after adjustment for additional clinical variables. In addition, we observed that the NFS was an independent risk factor for CKD in subjects with MAFLD with DM or prediabetes (OR 1.359, 95% CI 1.141–1.617, *p* = 0.001), similar to the results of Sun et al. [14]. Furthermore, a recent 4.6-year cohort study in China demonstrated that MAFLD increased the incident risks of CKD and CVD [15]. In addition, we also found that MAFLD patients with CKD had a higher degree of cirrhosis than those without CKD (data not shown), which was consistent with the results of Ciardullo et al. [16] These findings suggested that MAFLD and its related metabolic disorders might play important roles in the development and progression of CKD, and potential treatments might benefit both CKD and MAFLD.

Remnant cholesterol is defined as triglyceride-rich lipoprotein cholesterol and is calculated as total cholesterol minus HDL-C and LDL-C. Recent studies have confirmed that remnant cholesterol, not HDL-C or LDL-C, is significantly associated with an increased risk of CVD [17,18]. A higher level of remnant cholesterol was also related to the prevalence of CKD in a general middle-aged population [19] and was predictive of all-cause, cardiovascular-related, and cancer-related mortality in individuals with MAFLD [20]. In our study, we demonstrated that remnant cholesterol was independently related to an increased risk of CKD in all subjects after PSM, as well as in individuals with MAFLD with DM or prediabetes, which suggests that elevated serum remnant cholesterol might contribute to the development of CKD in subjects with MAFLD. According to these findings, screening and evaluation of remnant cholesterol can more accurately identify a high risk of CKD and prevent prevalent CKD. Further studies are needed to elucidate the value of remnant cholesterol monitoring in MAFLD management.

CKD is characterized by a state of low-grade inflammation [21], and visceral adipose tissue (VAT) is related to microinflammation [22,23]. Emerging evidence has indicated that VAT is a better predictor of the development of CKD than BMI [24,25]. Recently, Tsai et al. [26] reported that a higher BFP and higher high-sensitivity (hs) CRP level were related to renal dysfunction in a general population. In this study, we also observed that CRP was an independent risk factor for CKD in the general population, and BFP was significantly associated with the prevalence of CKD in subjects with MAFLD without DM. In addition, the BFP was positively correlated with CRP (*r* = 0.217, *p* < 0.001). These findings indicate that BFP may play an important role in renal injury in individuals with MAFLD with metabolic abnormalities, though this remains to be confirmed in additional studies.

Age, hypertension, DM, obesity, and dyslipidemia are common risk factors for CKD progression [27,28]. As predicted, the above covariates were also independently associated with the increased prevalence of CKD in this study, and several studies have shown that hypertension might be an important factor linking MAFLD and CKD [29]. The value of hyperuricemia in predicting CKD development in the general population is still controversial; however, it has been shown to be related to an increased prevalence of CKD in individuals with NAFLD and MAFLD [12,30]. We also found that hyperuricemia was significantly related to CKD in all individuals and subgroups, with a 2.036-fold higher risk than that in controls.

DM has become the major cause of CKD and ESRD in both developed and developing countries [31–33], and nearly 30–40% of affected individuals will develop renal injury [34]. In this study, we demonstrated that DM was an independent risk factor for CKD in the general population, similar to the results of Deng et al. based on the dataset of the NHANES 2017–2018 [12]. Interestingly, before PSM, individuals with MAFLD had a lower eGFR than those without MAFLD; however, the eGFR was higher in the MAFLD group than in the non-MAFLD group after PSM. This variation was most likely due to MAFLD-related metabolic dysfunction, such as DM and obesity, which contributed to glomerular hyperfiltration after adjustment for age, sex, and ethnicity. A similar reverse pattern was observed in the study by Deng et al. [12].

Liver–kidney crosstalk in MAFLD includes alterations in the renin-angiotensin system (RAS), insulin resistance and activated protein kinase (AMPK) activation, impaired antioxidant defenses, and excessive dietary fructose intake, which affects kidney injury by altering lipogenesis and inflammatory responses [35,36]. Moreover, the insulin resistance involved in MAFLD can in turn aggravate lipid metabolism disorders, forming a vicious cycle. Imbalances in inflammatory cytokines [37], the activation of oxidative stress [36,38], and insulin resistance [39,40], the involvement of the RAAS system [41], production of hypoadiponectinemia and atherogenic dyslipidemia [40] and disruption of the intestinal barrier resulting from gut dysbiosis [42,43], increases in uremic toxins and secondary bile acids, and decreases in short-chain fatty acids are considered to be the common pathophysiological mechanisms linking MAFLD and CKD [44].

To date, rare large studies on the pharmacological or nonpharmacological treatment of MAFLD patients with CKD have been reported [35]. However, because MAFLD and CKD share several common risk factors (e.g. obesity, insulin resistance, dyslipidemia, hypertension, and dysglycemia) and pathogenetic pathways, current management of MAFLD patients with CKD focuses on lipid and glucose lowering, blood pressure control, and weight loss [35]. Several studies have shown that sodium-glucose cotransporter-2 (SGLT2) inhibitors, peroxisome proliferator-activated receptor (PPAR) agonists, farnesoid X nuclear receptor (FXR) agonists, thyroid hormone receptor β (TR β) agonists and proprotein convertase subtilisin-kex in type 9 (PCSK9) inhibitors may be promising agents for the treatment of MAFLD patients with CKD [35,45]. Additionally, modulation of gut microbial components and mesenchymal stem cells (MSCs)-based therapy may be novel therapeutic strategies for MAFLD and CKD [46,47].

Several limitations in this study should be noted. First, it was a cross-sectional study in a single Chinese center, of which the findings might only apply to the Chinese population, and the diagnosis of CKD was based on a single examination of eGFR and UACR rather than several tests over three months. Second, the diagnostic criteria for overweight/obesity in China are BMI >24kg/m^2^, which is different from the criteria ‘BMI >23 kg/m^2^’ in this study. This might have a certain impact on the generalization of the results of this study in China. Third, given the limitation of datasets, some diagnostic indicators involved in MAFLD were unavailable, including the HOMA-IR score, hs-CRP level, the specific type of diabetes, the duration of diabetes, drinking status, hepatitis B virus infection or other liver disease. Fourth, the drug information of the subjects was lacking. Hence, we did not take therapeutic interventions into account, and other confounding factors, such as alcohol consumption, might affect the development of CKD. Fifth, the hepatic steatosis in this study was diagnosed based on FibroScan controlled attenuation parameter (CAP) ≥240 dB/m using liver ultrasound transient elastography, which may miss mild fatty liver, and biopsy data, which are specifically useful to assess liver fibrosis, were unavailable in this study.

In summary, our study demonstrated that MAFLD might affect the development of CKD, though it is not an independent risk factor. Further studies are needed to identify the role of MAFLD in the trajectory of CKD and whether it could alter the overall prognosis of CKD. In addition, given that individuals with MAFLD might be more prone to chronic kidney damage, more attention should be given to identifying those at high risk of metabolic disorders; this information will be helpful in further advancing the clinical management of MAFLD and CKD.

## Abbreviations

MAFLD: Metabolic Associated Fatty Liver Disease
NAFLD: Nonalcoholic Fatty Liver Disease
DM: Diabetes Mellitus
CKD: Chronic Kidney Disease
ALT: Alanine Aminotransferase
AST: Aspartate Aminotransferase
CRP: High Sensitivity C-Reactive Protein
UACR: Urine Albumin Creatinine Ratio
BMI: Body Mass Index
GGT: γ-Glutamyl Transpeptidase
ALP: Alkaline Phosphatase
HDL-C: High-Density Lipoprotein Cholesterol
LDL-C: Low-Density Lipoprotein Cholesterol
HbA1c: Hemoglobin A1c
FIB-4: Fibrosis 4
NFS: NAFLD Fibrosis Score
BFP: Body Fat Percentage
OR: Odds Ratio
